# Resistance Is Not Futile: Widespread Convergent Evolution of Resistance to Alpha-Neurotoxic Snake Venoms in Caecilians (Amphibia: Gymnophiona)

**DOI:** 10.3390/ijms241411353

**Published:** 2023-07-12

**Authors:** Marco Mancuso, Shabnam Zaman, Simon T. Maddock, Rachunliu G. Kamei, David Salazar-Valenzuela, Mark Wilkinson, Kim Roelants, Bryan G. Fry

**Affiliations:** 1 Amphibian Evolution Lab, Biology Department, Vrije Universiteit Brussel, Pleinlaan 2, 1050 Brussels, Belgium; 19marcomancuso19@gmail.com (M.M.); shabnam.zaman@vub.be (S.Z.); 2 Department of Life Sciences, The Natural History Museum, London SW7 5BD, UK; s.t.maddock@gmail.com (S.T.M.); rgkamei@fieldmuseum.org (R.G.K.); m.wilkinson@nhm.ac.uk (M.W.); 3 School of Life Sciences, University of Wolverhampton, Wulfruna Street, Wolverhampton WV1 1LY, UK; 4 Island Biodiversity and Conservation Centre, University of Seychelles, Mahé P.O. Box 1348, Seychelles; 5 Amphibians and Reptiles Division, The Field Museum of Natural History, 1400 S Lake Shore Dr., Chicago, IL 60605, USA; 6 Centro de Investigación de la Biodiversidad y Cambio Climático (BioCamb) e Ingeniería en Biodiversidad y Recursos Genéticos, Facultad de Ciencias de Medio Ambiente, Universidad Indoamérica, Machala y Sabanilla, Quito EC170301, Ecuador; davidsalazar@uti.edu.ec; 7 Venom Evolutionary Lab, School of Biological Sciences, University of Queensland, St. Lucia, QLD 4072, Australia

**Keywords:** nicotinic acetylcholine receptor, neurotoxin-binding, toxin resistance, evolution, Darwinian selection

## Abstract

Predatory innovations impose reciprocal selection pressures upon prey. The evolution of snake venom alpha-neurotoxins has triggered the corresponding evolution of resistance in the post-synaptic nicotinic acetylcholine receptors of prey in a complex chemical arms race. All other things being equal, animals like caecilians (an Order of legless amphibians) are quite vulnerable to predation by fossorial elapid snakes and their powerful alpha-neurotoxic venoms; thus, they are under strong selective pressure. Here, we sequenced the nicotinic acetylcholine receptor alpha-1 subunit of 37 caecilian species, representing all currently known families of caecilians from across the Americas, Africa, and Asia, including species endemic to the Seychelles. Three types of resistance were identified: (1) steric hindrance from N-glycosylated asparagines; (2) secondary structural changes due to the replacement of proline by another amino acid; and (3) electrostatic charge repulsion of the positively charged neurotoxins, through the introduction of a positively charged amino acid into the toxin-binding site. We demonstrated that resistance to alpha-neurotoxins convergently evolved at least fifteen times across the caecilian tree (three times in Africa, seven times in the Americas, and five times in Asia). Additionally, as several species were shown to possess multiple resistance modifications acting synergistically, caecilians must have undergone at least 20 separate events involving the origin of toxin resistance. On the other hand, resistance in non-caecilian amphibians was found to be limited to five origins. Together, the mutations underlying resistance in caecilians constitute a robust signature of positive selection which strongly correlates with elapid presence through both space (sympatry with caecilian-eating elapids) and time (Cenozoic radiation of elapids). Our study demonstrates the extent of convergent evolution that can be expected when a single widespread predatory adaptation triggers parallel evolutionary arms races at a global scale.

## 1. Introduction

In any predator–prey relationship, the animals most vulnerable to predation are under the strongest selective pressure to evolve a form of defense. This may be mechanical, such as the ability to run fast, or chemical, such as venom or poison. Amphibians have developed many potential defense mechanisms. For example, some species are explosive jumpers that are able to cover many body lengths in a single jump, often towards the water. Others (tree frogs) are gliders, capable of escaping arboreal predators or birds by parachuting down to safety. A wide range of frogs and salamanders are poisonous, with their granular skin glands either secreting endogenous toxins, toxins bioaccumulated from the diet, or, when associated with puncturing structures, even venom [1,2,3,4,5]. Although many caecilians may produce skin poisons too, there is remarkably little evidence [6]. Caecilians are found in the diets of many semi-fossorial venomous snakes [7,8,9,10,11,12]. Therefore, they might be under particularly strong pressure to evolve a resistance to snake venom.

Venom is a key evolutionary innovation underpinning the radiation of advanced snakes (giving rise to several families, including Elapidae) [13,14,15,16]. Correspondingly, venom resistance has evolved in the prey and predators of venomous snakes, as well as in the snakes themselves as resistance to their own venom. The resistance may involve diverse mechanisms and some species may possess more than one type. Documented mechanisms range from serum factors that intercept the venom’s toxins before they reach their pathophysiological targets, to modifications of the targets themselves to prevent docking by the toxins [17]. Serum factors conferring resistance to sympatric venomous snakes have been documented in rodents (rats, squirrels, and voles) to escape rattlesnake predators [18,19,20,21,22,23,24,25,26]; opossums (family Didelphidae) to against pit viper venoms (Crotalinae), which may be both prey and predators [27,28,29,30]; mongooses (*Herpestes* spp.) to act against the venom of snake prey [31]; the Boa Constrictor (*Boa constrictor*) to escape coral snake (*Micrurus* spp.) predators [32]; the Indigo Snake (*Drymarchon couperi*) to act against pit viper prey’s venom [33]; and in many venomous snakes against their own toxins [34,35]. Modifications of the target itself include von Willebrand factor (VWF) in opossums, which acts against the venoms of pit viper prey and predators [36]. Modifications of the post-synaptic acetylcholine receptor that act to confer resistance to snake alpha-neurotoxins have evolved on a myriad of occasions, including in mongooses against the venom elapid snake prey [31]; in diverse reptiles (Burmese Python (*Python bivittatus*), Mole Snake (*Pseudaspis cana*), Savannah Monitor Lizard (*Varanus exanthematicus*)) and amphibians (caecilians) against the venom of elapid snake predators [37,38,39]; in primates [40] as part of an evolutionary arms race with sympatric cobras [41]; and in neurotoxic snakes against their own venom [39,42]. In some cases, resistance has been documented but it is unclear if it is due to serum factors or changes in the toxin target itself. One example is the resistance of the Australian Eastern Blue-Tongue Skink (*Tiliqua scincoides*) against the venom of the sympatric predator the Red-belly Black Snake (*Pseudechis porphyriacus*), whereby the procoagulant toxins in the venom are unable to activate prothrombin (Factor II) in the skink’s blood and cause lethal coagulopathy [43].

Snake venom neurotoxins act at diverse sites within the neuromuscular junction [44]. Processes occurring at the pre-synaptic end either involve blocking pathophysiological targets such as ion channels (calcium, potassium, sodium), or, alternatively, causing physical damage to the nerve terminals. Reciprocally, post-synaptic neurotoxicity acts upon muscarinic receptors and nicotinic acetylcholine receptors on skeletal muscle. The most widely distributed neurotoxic trait across snakes is the ability to block the endogenous neurotransmitter acetylcholine from binding to the 14-residue-domain orthosteric site (amino acids 187–200 of the post-synaptic nicotinic acetylcholine receptor’s alpha-1 subunit, encoded by the gene *chrna1*). The inability of acetylcholine to bind and trigger normal muscle contraction results in muscle paralysis, eventually affecting the diaphragm, leading to death by respiratory failure [15,45]. As noted above, the interactions between alpha-neurotoxic snakes and their prey, as well as their predators, have led to the recurrent evolution of resistance to these toxins. Three different mechanisms have been identified to date, all of which occur at the orthosteric site within the alpha-1 subunit: N-glycosylation of an asparagine residue (N) [46,47,48,49,50], substitutions of a proline residue [31,51,52], and electrostatic charge-repulsion [37].

**Figure 1:**
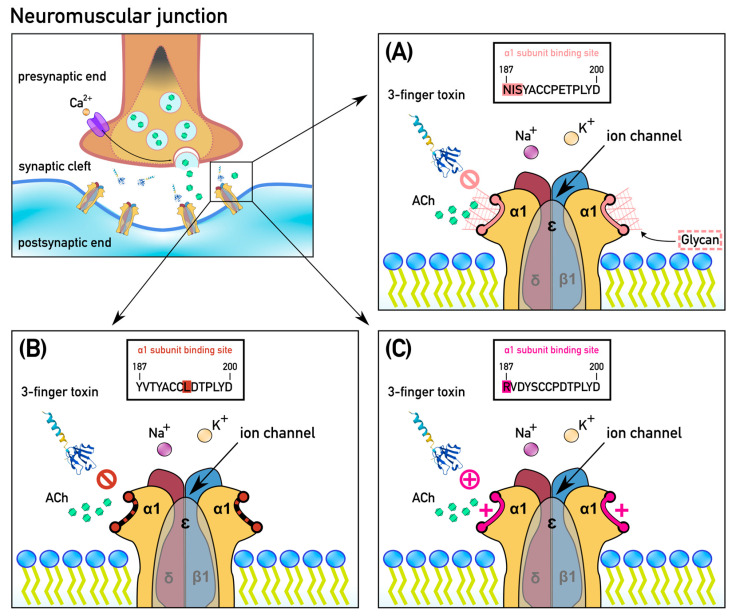
Modifications to the post-synaptic nicotinic acetylcholine receptor (nAchR) conferring resistance to alpha-neurotoxins. (**A**) N-glycosylation at an NX(S/T) motif (at positions 187–189 or 189–191). This physical form of resistance creates spatial congestion, preventing larger molecules, such as alpha-neurotoxins, from reaching the binding site, while allowing smaller molecules, such as the neurotransmitter acetylcholine, to still reach their destination. (**B**) Substitution of proline residues at positions 194 or 197. This substitution creates a conformational change in the substrate and reduces toxin affinity to the receptor. (**C**) Introduction of a positively charged amino acid (arginine (R) or lysine (K)), which causes electrostatic repulsion of alpha-neurotoxins that are themselves also positively charged.

N-glycosylation is conferred by changes in the amino acid sequence: the tripeptide motif NX(S/T) emerges so that asparagine is followed by any amino acid (except cysteine (C) or proline (P)) which is, in turn, followed by either serine or threonine [46,47,48,49,50]. N-glycosylation involves the asparagine’s nitrogen molecule being linked to a glycan consisting of a carbohydrate backbone with branching sugar chains. This post-translational modification blocks the docking of the neurotoxins through steric hindrance, whereby the orthosteric site is congested by shrubbery-like sugar chains while the much smaller endogenous neurotransmitter acetylcholine is still able to penetrate and stimulate muscle contraction (Figure 1A). N-glycosylation was first documented in mongooses to confer resistance to cobra venom, with the glycosylation occurring to an asparagine at position 187 of the orthosteric site [31,51,52]. Subsequently, it was shown to have evolved convergently within cobras as resistance to their own venom; however, this involved an asparagine at position 189 [42]. N-glycosylation was later documented in a wide range of additional neurotoxic venomous snakes, as well as within both their predators (such as meerkats, revealing alpha-neurotoxin-resistance as a trait shared in their last common ancestor with mongooses) and prey (such as the slow-moving Eastern Bearded Dragon (*Pogona vitticeps*)) [39].

The second mode of resistance, proline substitution, involves a change in the secondary structure of the orthosteric site. Proline residues, such as those evolutionarily conserved at positions 194 and 197 within the alpha-1 orthosteric site, induce a kink in an amino acid chain, thereby conferring a discrete type of secondary structure (Figure 1B). The substitution of these prolines by other amino acids has been shown to reduce the binding of snake venom alpha-neurotoxins [31,51,52]. This mutation has arisen multiple times within the prey of elapid snakes [37,39].

The most recently discovered form of the inhibition of neurotoxin-binding is that of electrostatic charge repulsion, whereby a positively charged amino acid is introduced into the orthosteric sequence in place of a negatively charged amino acid. This mechanism repels the snake venom neurotoxins, which display strongly positively charged molecular surfaces [37]. The orthosteric site typically contains a negatively charged amino acid (aspartic acid (D) or glutamic acid (E)) at either position 191 or 195 (or both), thus conferring an inherent negatively charged state to the orthosteric site. This adaptation has, in turn, exerted a selection pressure for alpha-neurotoxins to evolve with strongly positively charged molecular surfaces, thereby facilitating the docking of the toxin via an opposite-charge attraction effect [53]. Mutations that reverse the charge at one or both positions by encoding for a positively charged amino acid (such as lysine (K)) confer resistance as the neurotoxins are now electrostatically repulsed by the same-charge interaction, analogous to the repelling effect of attempting to bring the same sides of two magnets together [37] (Figure 1C). This has been documented in slow-moving terrestrial animals that may be preyed upon during their early life stages, such as the Burmese Python (*Python bivittatus*) and the Mole Snake (*Pseudaspis cana*) [37]. It has additionally been shown that the substitution of negatively charged amino acids at positions 191 and 195 with uncharged amino acids also confers a significant level of resistance (but less than that of substitution by a positively charged amino), as seen in the Savannah Monitor Lizard (*Varanus exanthematicus*), another potential prey item in the diet of true cobras (*Naja* spp.) [38]. In the Honey Badger (*Mellivora capensis*), a major predator of neurotoxic elapid snakes, the substitution of uncharged tryptophan (W) with the positively charged arginine (R) at position 187 [54] has been shown to be entirely responsible for their famed resistance to cobra venoms [37].

In a comprehensive study examining the convergent evolution of alpha-neurotoxin resistance in vertebrates, it was noted that two out of three caecilian species examined contained one or more toxin resistance motifs in the orthosteric site [39]. *Microcaecilia unicolor* (from the South American family Siphonopidae) and *Rhinatrema bivittatum* (from the South American family Rhinatrematidae) both contain an arginine at position 187. Additionally, *R. bivittatum* also features a proline substitution at position 194. In contrast, the African species *Geotrypetes seraphini* (Dermophiidae) lacked any known form of resistance. This first reported evidence of toxin resistance in caecilians is intriguing because of their physical characteristics and ecological niche. Most caecilians are fossorial (except for the South American family Typhlonectidae, which is secondarily aquatic), legless, worm-like amphibians [55,56]; therefore, they are thought to be vulnerable to sympatric fossorial-hunting elapids across their ranges, particularly in the Americas (e.g., *Leptomicrurus* and *Micrurus*) [7,8,9,10,11] and Asia (e.g., *Bungarus*, *Calliophis*, *Naja*, and *Sinomicrurus*) [57,58,59,60].

Caecilian diversification patterns and their current distribution reflect a Pangaean origin with subsequent Gondwanan radiation [61,62,63]. Elapid snakes are the dominant venomous snake predators in the fossorial niche and have been documented as preying on caecilians [7,8,9,10,11]. We hypothesized that caecilians are therefore under strong selective pressure to evolve resistance to elapid venom. As caecilians spread geographically prior to the evolution of elapid snakes, we further hypothesized that the examination of additional species would reveal evidence of widespread convergent evolution. To test this hypothesis, we examined the gene fragment encoding the orthosteric site of the alpha-1 subunit of 40 caecilian species belonging to all 10 currently recognized families [56,63,64,65,66], as well as 24 non-caecilian amphibians. In addition to reconstructing the evolution of experimentally validated toxin resistance motifs, we investigated patterns of positive selection as a function of elapid sympatry.

## 2. Results

### 2.1. Extensive Convergent Evolution of Motifs of Resistance to Alpha-Neurotoxins

**Figure 2:**
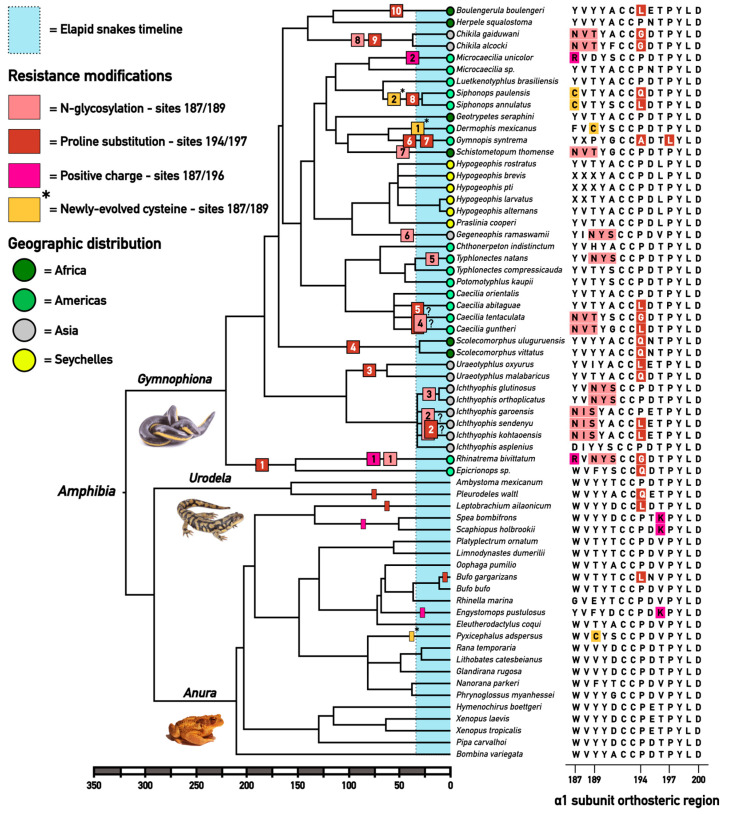
Caecilian orthosteric site mutations linked to alpha-neurotoxin-resistance mapped on the amphibian phylogeny, with a scale bar representing millions of years of diversification. The initial tree was obtained from Timetree.org [68] and has been adapted to include caecilians belonging to the family Chikilidae, following Kamei et al., 2012 [56]. Phylogenetic ambiguities at internal nodes are represented as soft polytomies. Character history was traced using Mesquite (v.3.80.) [69] by unordered maximum parsimony under the accelerated transformation (Acctran, favoring changes occurring as close as possible to the root). Boxes representing the evolution of resistance motifs are arbitrarily placed at the median point of the branch where they occur. Colored boxes are numbered to represent the number of origins for each resistance motif within caecilians. The novel evolution of a cysteine at position 187 or 189 (light orange) is displayed with an asterisk, as functional tests are required to assess the role of this modification in conferring toxin-resistance. Question mark labels are placed next to the resistance origins occurring at polytomies in order to indicate uncertainty regarding the branch within the clade along which the motif actually arose.

The in silico translation of 40 caecilian *chrna1* orthosteric domains, including 37 new sequences obtained through locus-specific PCR and Sanger sequencing (see Methods for details), reveal a large proportion of caecilian species with motifs hindering the binding of alpha-neurotoxins. The results indicate at least 20 evolutions of resistance modifications. As some lineages evolved more than one form of resistance, this resulted in at least 15 events of convergent evolution of toxin-resistance in caecilians (Figure 2). The most common was the substitution of a proline, found in 17 out of the 40 examined species, followed by NX(S/T) motifs, which were found in 13 species. The evolution of a positively charged amino acid was observed in only two species. Eight species had more than one toxin resistance motif, typically combining a proline substitution with an NX(S/T) motif.

To reconstruct the evolution of these observed toxin resistance motifs, we aligned the orthosteric site sequences and estimated ancestral character states on a consensus phylogeny of caecilians (see Methods and Figure 2). For comparison, we expanded the data set and corresponding tree to include 24 non-caecilian amphibian sequences (anuran and urodelan) retrieved from online databases [67]. According to the most parsimonious reconstruction, toxin resistance motifs evolved at least 20 times in caecilians (Figure 2). The actual number of origins may be higher and/or some motifs may have been secondarily lost depending on (1) the optimization method (accelerated vs. delayed transformation, respectively favoring successive or parallel state changes in cases of ambiguity; see Methods) or (2) how remaining polytomies in the consensus tree are resolved. In contrast, toxin resistance motifs were noted to have only evolved five times across other amphibians, occurring in the forms of proline substitutions (three origins) and electrostatic repulsion (two origins), resulting in a total of six non-caecilian species with modifications to the orthosteric site of the alpha-1 subunit.

N-glycosylation is believed to be the most effective process of preventing toxins from docking to the receptor, forming a physical barrier that impedes binding to the orthosteric site [39,46,47,48,49,50,52]. The required NX(S/T) motif evolved on at least eight occasions: four times at positions 187–189 and four times at positions 189–191 (light pink labels in Figure 2). The motif also evolved independently on different continents: once in Africa (in the genus *Schistometopum*), three times in America (in the genera *Rhinatrema*, *Caecilia,* and *Typhlonectes*), and four times in Asia (once in the genera *Gegeneophis* and *Chikila* and twice at different positions in the genus *Ichthyophis*). These numbers are roughly consistent with the reports of the elapid predation on caecilians in Asia and America ([7,8,9,10,11,57,58,59,60] *sensu lato*) and the apparent lack thereof in Africa. The motifs at positions 187–189 all have an isoleucine (I) or valine (V) as the second residue; meanwhile, those at position 189–191 share an evolutionarily conserved tyrosine (Y) residue. Interestingly, in some reconstructed cases, the motif may have evolved in a stepwise manner. For example, while the C-terminal serine of the motif appeared at position 191 in an ancestor of *Typhlonectes* and *Potomotyphlus*, the asparagine at position 189 originated only later in *Typhlonectes natans*, completing the motif. This pattern suggests that the origin of the NX(S/T) motif is not necessarily explained by natural selection for toxin-resistance alone (but see further). Instead, historical contingency may have facilitated its convergent appearance across multiple lineages.

Proline substitutions modified the orthosteric site’s secondary structure at least ten times: nine times at position 194 (Figure 2) and once at position 197 (in *Gymnopis*). As the secondary structure imposed by ancestral prolines at both positions has been shown to be necessary for the complete binding of alpha-neurotoxins, the substitution of this ancestral residue lowers the affinity of the toxin to the receptor. We found proline to be replaced by: leucine (L) in seven caecilian species (*Boulengerula boulengeri* [Herpelidae], *Siphonops annulatus* [Siphonopidae], *Caecilia abitaguae*, *C. guntheri* [Caeciliidae], *Uraeotyphlus oxyurus*, *Ichthyophis sendenyu*, *I. garoensis* [Ichthyophiidae]); by glutamine (Q) in five species (*Siphonops paulensis* [Siphonopidae], *Scolecomorphus uluguruensis*, *S. vittatus* [Scolecomorphidae], *Uraeotyphlus malabaricus* [Ichthyophiidae], *Epicrionops sp.* [Rhinatrematidae]); by glycine (G) in four species (*Chikila alcocki*, *C. gaiduwani* [Chikilidae], *Caecilia tentaculata* [Caeciliidae], *R. bivittatum* [Rhinatrematidae]); and by alanine (A) in one species (*Gymnopis syntrema* [Dermophiidae]) (Figure 2). In addition, proline substitution also occurred at least three times in non-caecilian amphibians: once in salamanders (as testified by *Pleurodeles waltl*) and twice in frogs (as shown by *Bufo gargarizans* and *Leptobrachium ailaonicum*).

Mutations resulting in electrostatic charge repulsion were rarer across caecilians, with only two origins at position 187 reconstructed in distinct lineages from the Americas. Interestingly, substitutions introducing a positively charged lysine (K) residue at position 196 were identified in two frog lineages: Neotropical Leptodactylidae (represented by *Engymostops pustulosus*) and North American Scaphiopodidae (represented by the genera *Scaphiophus* and *Spea*).

Combined, the evolution of the three types of toxin resistance motifs in caecilians seems to show differential patterns across continents. Overall, we recover three origins in African lineages (retained in four analyzed African species), ten origins in American lineages (retained in ten American species), and seven origins in Asian lineages (retained in ten Asian species). The NX(S/T) motif evolved once in Africa (in *Schistometopum*), three times in America (in *Rhinatrema*, *Caecilia*, and *Typhlonectes*), and four times in Asia (once in *Gegeneophis* and *Chikila* and twice at different positions in *Ichthyophis*). Similarly, proline substitutions occurred twice in Africa (in *Scolecomorphus* and *Boulengrula*), five times in America (once in *Siphonops*, *Caecilia,* and *Rhinatrema* and twice in tandem in *Gymnopis*), and three times in Asia (in *Chikila*, *Ichthyophis*, and *Uraeotyphlus*). The two reconstructed origins of electrostatic charge repulsion both occurred in American caecilians (*Microcaecilia* and *Rhinatrema*). Notably, no toxin resistance motifs were found in the caecilian clade endemic to the Seychelles archipelago (*Hypogeophis* and *Praslinia*), where elapid snakes are historically absent.

In addition to suggesting a spatial correlation of motif evolution with elapid presence, our reconstruction also seems to imply a temporal overlap. Most origins of the toxin-resistance motifs are reconstructed on relatively recent branches. When the amphibian phylogeny is presented as a time tree (reflecting estimated divergence times; Figure 2) many motif origins are reconstructed along terminal branches (extending to the present time) or along ancestral branches that lie at least partially in the Cenozoic. Consequently, they overlap with the radiation of elapid snakes, which started at the beginning of the Oligocene [16].

### 2.2. Comparative Selection Analyses Indicate Strong Adaptive Pressure to Neurotoxin-Resistance in American and Asian Caecilians

Our analyses identify an intensified burst of mutations in a neurotoxin-binding site in line with a large-scale adaptive response of caecilians to envenomation by Elapidae. We examined the extent to which the observed pattern was driven by positive selection for toxin resistance by conducting a series of comparative selection analyses using the software package pamlX (v.1.3.1) [70]. These analyses involved estimating the ratio of non-synonymous over synonymous substitutions (ω) in the underlying codons in caecilians and other amphibians of the orthosteric site, its flanking regions, and its paralogous regions in other receptor subunits (see Methods).

First, analysis of a data set of codon sequences encoding the orthosteric sites of the 24 non-caecilian amphibians shown in Figure 2 computed an ω of 0.0852 (Table 1). This value suggests very strong purifying selection, as can be expected for an evolutionarily conserved neural receptor. An equivalent analysis of the codon sequences of the orthosteric sites of the 40 caecilians returned an ω of 0.6019. Although smaller than an ω of 1, this value suggests that either the orthosteric site in caecilians undergoes overall more relaxed selection than in non-caecilian amphibians or it represents a mixture of codon positions under purifying and positive selection. To examine how this pattern correlates with the presence of elapid snakes, we next applied a selection model that estimated separate ω values for caecilian branches that overlapped in time with elapid presence vs. branches that did not (a so-called ‘branch model’ in pamlX). Examples of the latter include ancestral branches in the time tree (Figure 2) that are older than the origin of Elapidae [16,71]) or branches endemic to the Seychelles, where elapids likely never occurred (see Methods and Supplementary Figure S1). This analysis strengthens the previously observed pattern: while caecilian branches that evolved in the absence of elapid snakes share an estimated ω value of 0.2168 (strong purifying selection), those that evolved at least partially in the presence of elapids show an ω value of 0.9699 (nearly neutral evolution or a mixture of purifying and positive selection). Similarly, to examine the geographic variation in selective regimes across caecilians, we applied a model that estimated distinct ω values for branch sets divided per land mass (Africa, America, Asia, and the Seychelles; see Methods and Supplementary Figure S2). This analysis suggests purifying selection along ancestral branches older than the elapid radiation [16,71], African branches, and Seychelles branches (ω = 0.2411, 0.1703, and 0.0001, respectively). In contrast, American and Asian branches are shown to have evolved under positive selection (ω = 1.7789 and 2.6121, respectively), indicating major parallel shifts in the selective regimes in these continents. To investigate which specific positions in the orthosteric site are involved in this shift, we conducted an analysis that investigates codon-specific changes in the selection between background and foreground branch sets (a so-called ‘branch-site model’ in pamlX). This analysis indicates that four of the fourteen codons encoding the orthosteric site changed from a regime of purifying selection or neutral evolution to strong positive selection (ω = 12.7621) in Asian and American lineages. These codons correspond to the first and last positions of the two NX(S/T) motifs (positions 187, 189, and 191) and the position at which most proline substitutions have been observed (position 194).

If the above-mentioned positive selection indeed reflects natural selection for toxin resistance (and not the adaptive evolution of a larger part of the alpha subunit driven by another unknown factor), we expect it to be restricted to the *chrna1* gene segment encoding the orthosteric (toxin-binding) site. We examined this by performing an equivalent selection analysis on the orthosteric site’s flanking regions that are not involved in toxin binding (the codon sequences encoding amino acids at positions 180–186 and 201–207). In contrast to the orthosteric sites of American and Asian lineages, the flanking regions show evidence of purifying selection in both lineages (ω = 0.4039 and ω = 0.7442, respectively; Table 1). Similarly, selection analyses performed on the homologous gene segments of two related subunits (alpha-2 and alpha-4, encoded by the genes *chrna2* and *chrna4*, respectively; see Methods) indicated a strong purifying selection for both caecilians and non-caecilian amphibians (all ω < 0.005; Table 2). This is consistent with the notion that alpha-neurotoxins only target the orthosteric site of the alpha-1 subunit, as paralogous subunits are *quasi*-inaccessible through the circulatory system [45].

Together, our analyses imply that positive selection is largely restricted structurally (to the toxin-binding site), physiologically (to the only bloodstream accessible receptor subunit), and geographically (to Asian and American caecilians). The latter is consistent with the geographic variation in the reconstructed numbers of motif origins (see previous section) and indicates that the orthosteric site of caecilians inhabiting Asian and American tropical regions have been under adaptive pressure to acquire toxin resistance in the face of elapid predation.

## 3. Discussion

The present study identifies a large-scale adaptive response against envenomation by elapid snakes in an ancient amphibian clade. Although this pattern involves three previously documented motifs known to at least partially inhibit the binding of alpha-neurotoxins, its extent is currently unreported for any other vertebrate group apart from snakes [39]. As N-glycosylation is believed to be the most effective way of preventing toxins from docking to the receptor [39,46,47,48,49,50,52], it is logical that the NX(S/T) motif enabling this post-translational modification evolved so many times and at different positions. Yet, the substitution of prolines at positions 194 and 197, which alters the secondary structure of the orthosteric site, was found to be the most recurrent mutation reducing toxin binding. While the effects of leucine and serine as proline substitutes are well-documented [31,51,52], substitution with other amino acids is less understood. However, the physicochemical nature of alanine resembles that of leucine as both have a hydrophobic side chain; meanwhile, glutamine is similar to serine in having an uncharged polar side chain. The effect of glycine replacing proline remains unknown, but as it represents the replacement of a kink-inducing amino acid by the smallest possible amino acid, we hypothesize that it reduces toxin affinity to the receptor as well. To assess how various residues at positions 194 and 197 affect the binding of alpha-neurotoxins, functional experiments on mutant receptors will be required. However, it is noteworthy that many of the proline substitutions occur in combination with NX(S/T) motifs (e.g., in *Chikila*, *Caecilia*, *Ichthyophis*, and *Rhinatrema*), a pattern which has been shown to have a greater impact on toxin binding than single-site replacements [39,52].

Apart from the three previously established toxin resistance motifs, our analyses also reveal other mutations in the orthosteric sites of caecilians. One example that stands out is the origin of a cysteine (C) residue at positions 187 (in the last common ancestor of *Siphonops annulatus* and *Siphonops paulensis* [Siphonopidae]) and 189 (*Dermophis mexicanus* [Dermophiide]) (Figure 2). If this newly evolved cysteine is capable of forming a disulfide bond, it could radically change the subunit’s tertiary structure and the shape of the orthosteric site, affecting the binding ability of neurotoxins. Sequencing of the full alpha subunit to identify its possible partner cysteine, as well as structure-activity analyses to examine the impact of this mutation on neurotoxin binding, could test the hypothesis that an extra disulfide bond may represent a novel form of toxin resistance. If this modification is confirmed to decrease toxins’ ability to bind to the receptor, the evolution events across the caecilian tree would, therefore, increase to a staggering 22 independent occasions just within caecilians.

The multiple convergent origins of resistance motifs in Asian and American caecilians and the corresponding signature of positive selection are in line with reports of caecilian susceptibility to elapid predation in these regions ([7,8,9,10,57,58,59,60], *sensu lato*). Both continents are inhabited by multiple caecilian lineages, rather than a single clade, implying that the observed adaptive evolution is not determined phylogenetically; rather, it is determined geographically. *Ichthyophis* spp. (Ichthyophiidae), *Chikila* spp. (Chikilidae), and *Gegeneophis* spp. (Grandisoniidae) are three distinct lineages, all of which inhabit the Indian subcontinent and one of which (Ichthyophiidae) dispersed further into Southeast Asia. Each of these lineages has previously been hypothesized to have arrived in Asia by inhabiting the Indian subcontinent after its breakup from the rest of Gondwana [56,72]. Nevertheless, as India collided with the Asian mainland in the early Cenozoic [73,74,75,76], allowing faunal exchange between both land masses, each of the three caecilian lineages could have been exposed to elapid predation since the early history of these venomous snakes. In America, four caecilian lineages had to adapt in parallel to the arrival of elapids: Rhinatrematidae; a clade combining Caeciliidae and Typhlonectidae; American Dermophiidae; and Siphonopidae. This arrival took place via the dispersal of coral snakes from Asia, either by oceanic dispersal across the Pacific or via the Bering Strait, as shown in other reptilian lineages [76,77]. Based on molecular estimates for the divergence time of Asian *Sinomicrurus* and American coral snakes, this dispersal event most likely took place in the Oligocene, ≤ 30 Mya [16,71].

African caecilians, consistent with the absence of elapid snake predation reports, show no evidence of positive selection. Although a considerable diversity of elapid snakes lives in sympatry with African caecilians, there is no documented evidence of predation, possibly because these snakes have not occupied the same semi-fossorial niches as coral snakes did in Asia and the Americas. Instead, one study reports predation on the caecilian *Scolecomorphus kirkii* by the snake *Atractaspis aterrima* (Atractaspididae) [12], a semi-fossorial snake in a separate family (Atractaspididae) that is relatively closely related to Elapidae [16]. Although this snake is known to produce alpha-neurotoxins, its venom shows mainly hemotoxic action [78,79].

The presence of an NX(S/T) motif in *Schistometopum thomense* is intriguing. This species is thought to be endemic to São Tomé, an island in the Gulf of Guinea, although its presence in mainland Africa was documented by a specimen in Congo [80]. São Tomé is inhabited by only one elapid snake species: the recently described *Naja peroescobari* [81]. As this elapid snake is likely to have been introduced by Portuguese colonizers only in the 18th century [81], an arms race triggering the evolution of resistance in *S. thomense* on São Tomé is unlikely. An earlier mainland evolution of the trait is a more likely scenario, which may be resolved by future work examining species from the East African mainland, such as *Schistometopum gregorii* (which was unfortunately not available for inclusion in this study).

Caecilians in the genera *Hypogeophis* and *Praslinia* represent a single clade endemic to the Seychelles, a Gondwanan microcontinent that, together with India and Madagascar, separated from Africa and Antarctica in the Mid to Early Cretaceous [74,82] and finally separated from the Indian subcontinent around 65 Mya, prior to the latter’s collision with Asia (which may have occurred anywhere between 70 Mya and 35 Mya [75,76]). Consequently, the Seychelles were an isolated archipelago before the origin of elapids; therefore, caecilians endemic to the area have never faced any alpha-neurotoxin-bearing snakes throughout their history. In this light, it is not surprising that the Seychelles species all lack any of the known resistance motifs and share an orthosteric site that has remained under purifying selection, which is consistent with the continental species evolving under strong selection pressure due to the radiation of elapid snakes.

The observed pattern of large-scale adaptation in caecilians is currently unreported for any other vertebrate group besides the snakes themselves, as toxin resistance was found to have convergently evolved at least 15 times across this group of amphibians. However, it is unlikely that caecilians are entirely unique in having undergone this process. Even within non-caecilian amphibians, many lineages (e.g., Bufonidae) sharing geographic regions and habitats with Elapidae may have evolved similar adaptations. Among the other amphibians examined in this study, the frog species *Bufo gargarizans* (Bufonidae) and *Leptobrachium ailaonicum* (Megophryidae), as well as the salamander *Pleurodeles waltl* (Salamandridae), have undergone a proline substitution at the same position as caecilians and may show at least some resistance to alpha-neurotoxins. *B. gargarizans* is a East Asian toad that lives in sympatry with amphibian-eating cobra species [83]. *L. ailaonicum*, on the other hand, inhabits montane environments within a small range in the Yunnan region of China and, therefore, may be subject to predation from snakes of the elapid genera *Bungarus* or *Sinomicrurus* [84,85,86]. *Pleurodeles waltl* is a newt species known for its ability to envenomate predators by protruding its sharp ribs through its own skin glands. No elapids occur in its distribution range (the Iberian Peninsula and a small region in coastal Morocco); however, closely related *Pleurodeles* species occur in North Africa, possibly in sympatry with the Egyptian cobra *Naja haje*. Although this feeding generalist is known to prey on amphibians such as toads, it is unclear whether they pose a predatory threat to newts. Alternatively, *Pleurodeles* newts may have undergone a proline substitution to attain resistance to their own venom: a phenomenon that has previously been documented in several species of elapid snakes [39,42]. Testing this hypothesis, however, will have to await future characterization of *Pleurodeles* venom.

Mutations resulting in electrostatic charge repulsion also occurred in two anuran lineages, as testified by lysine residues in the South American frog *Engystomops pustulosus* (Leptodactylidae) and in the North American sister genera *Scaphiophus* and *Spea* (Scaphiopodidae). Although the predatory pressure on these taxa imposed by elapids is unknown, they partially occur in sympatry with coral snake species of the genus *Micrurus*. Modifications at position 196 were previously recorded in many snakes within the Elapoidea superfamily [37]. Our study represents the first documented instances of this resistance motif in amphibians. Further analyses could elucidate how widespread this phenomenon is across non-caecilian amphibians.

The results of multiple evolutions of alpha-neurotoxin resistance in caecilians is congruent with elapid snake neurotoxins being particularly potent on amphibians [87] and underscores the Red Queen type interaction between venomous predators and their prey. We anticipate that other, currently unstudied vertebrate taxa with similar geographic and ecological overlap with elapid snakes may show similar levels of convergent evolution in toxin resistance motifs. Together, these taxa would complete a worldwide picture in which the rise and dispersal of a single venomous predator group, elapid snakes with their potently alpha-neurotoxic venoms, had a strong impact on the evolution of multiple vertebrate prey taxa. 

## 4. Materials and Methods

### 4.1. Taxon Selection

Sequences from the orthosteric site of the alpha-1 subunit of the *chrna1* gene were obtained from 37 new caecilian species (Table 3). Tissue material for Sanger sequencing was kindly provided by different museum collections: BNHS (Bombay Natural History Society, Mumbai, India), CAS (California Academy of Sciences, Department of Herpetology, Golden Gate Park, San Francisco, CA 94118, USA), NHM (Natural History Museum, London, UK), CHUNB (Coleçao Herpetologica de Universidade Brasília, Brasília, D.F., Brazil), CP (Museu de Ciências e Tecnologia da PUCRS, Porto Alegre, Brazil), UMMZ (Museum of Zoology, University of Michigan, MI, USA), MZUTI (Universidad Indoamérica, Quito, Ecuador), WHT (Wildlife Heritage Trust, 95 Cotta Road, Colombo 8, Sri Lanka), and ZRC (Zoological reference collection, Lee Kong Chian Natural History Museum Faculty of Science, National University of Singapore, 2 Conservatory Drive, Singapore 117377). Species belonging to all ten currently recognized families of caecilians [56,63,64,65,66] are included in the present study. Taxa de novo sequenced were chosen based on both phylogenetic and geographic diversity.

### 4.2. DNA Extraction and Sequencing

DNA was extracted through a spin column protocol using the QIAGEN DNeasy Blood and Tissue Kit (QIAGEN, Carlsbad, CA, USA), according to the manufacturer’s protocols. Briefly, up to 25 mg of soft liver tissues were sliced thoroughly on Petri dishes with a scalpel, mixed in 2 mL Eppendorf tubes in a solution with Proteinase K and a lysis buffer, and then digested on a shaking water bath at 54 °C for lysis. Subsequently, lysates underwent a series of centrifugation steps in different buffer solutions for membrane-binding, washing, and final elution. Extracts were examined for purity (OD260/280 and OD260/230) and concentration (ng/μL) on a NanoDrop spectrophotometer (ThermoFisher Scientific, Waltham, MA, USA). Extracted DNA was stored at −20 °C. Aliquots to be used for PCR were maintained at 4 °C to avoid the effect of frequent freezing and thawing cycles.

A polymerase chain reaction (PCR) was used to amplify the sequence coding for the orthosteric site of nAChR, as previously successfully used by [39]. *Chrna1* is the gene coding for the alpha-1 subunit; thus, five *chrna1* amphibian sequences (*Rhinatrema bivittatum*, *Microcaecilia unicolor*, *Geotrypetes seraphini* (Table 3), *Nanorana parkeri*, and *Xenopus tropicalis* (Supplementary File S1)) were downloaded from NCBI GenBank [67] for primer design. NCBI Genome Data Viewer and the translate tool from Expasy [88] were used to identify exons encoding the region of interest within the alpha-1 subunit. The corresponding sequence was included in alignments with the other *chrna* sequences in Mafft (v.7.490.) [89]. Homologous sequences from paralogous *chrna* genes were downloaded from NCBI GenBank and included in the alignment used for primer design to ensure specificity for *chrna1* only. Candidate sequences were analyzed using SigmaAldrich OligoEvaluator software (http://oligoevaluator.com; accessed on 9 April 2021), under default settings, in order to design primers showing the lowest possible risk of forming secondary structures or dimerization. The following two newly designed locus-specific primers passed this quality control protocol: For1: GAAAGATTACCGAGGCTGGAAG, Rev1: CAAGGAATGATGACATTCACAAT; For2: GTGGAGAGTGGGTAATGAAAGATT, Rev2: CAAGGAATGATGACATTAACAAT. The annealing temperature for the PCR was set at 58 °C for the primer pair For1–Rev1 and at 60 °C for For2–Rev2 The expected amplicon size from the For1–Rev1 pair was 132 base pairs (bp) and 144 bp for For2–Rev2. The PCR reactions were carried out either as 50 μL reactions using Recombinant Taq DNA Polymerase (Invitrogen, Waltham, MA, USA) or 25 μL reactions using MyTaqTM Red Mix (Meridian Bioscience, Nottingham, UK), following the respective manufacturer’s instructions. The effectiveness and specificity of the PCR procedures were verified by agarose gel electrophoresis (2% agarose gel; 100 V for 65 min in TBE buffer).

The purified PCR products were outsourced for Sanger sequencing to BaseClear BV (Leiden, Netherlands) or sequenced in-house at University of Wolverhampton. The resulting electropherograms were inspected in CodonCode Aligner (v.9.0.2.; CodonCode Corporation, Centerville, MA, USA; http://www.codoncode.com/aligner/; accessed on 31 March 2022). Quality-checked and trimmed forward and reverse sequences were aligned in Mafft (v.7.490) [89] to elucidate ambiguous sites and compute the consensus sequence for each taxon. Nucleotide BLAST [90] homology searches against the NCBI GenBank were used to confirm the identity of each sequence as part of the *chrna1* gene.

### 4.3. Evolutionary Reconstruction of Toxin Resistance Motifs

The sequences of the 37 caecilians obtained by Sanger sequencing (see above) were combined with the orthologous sequences of three additional caecilians and 24 non-caecilian amphibians (two salamanders and 22 frog species). These were retrieved from the online databases of NCBI GenBank [67], Sal-Site [91], and iNewt [92] (Supplementary File S1). The sequences were aligned with Mafft (v.7.490) using the E-INS-i algorithm [89]. The alignment, containing 64 sequences, was subsequently divided into 2 equally large data sets of 42 nucleotides (or 14 codons): 1 corresponding to the orthosteric site (positions 187–200 of the alpha-1 subunit) and 1 corresponding to its flanking regions (by concatenating the 21 nucleotide positions left and right of the orthosteric site, i.e., positions 180–186 and 201–207).

The orthosteric site data set was translated *in silico* into amino acid sequences and used to infer the evolution of toxin resistance motifs via maximum parsimony character reconstruction with the program Mesquite (v.3.80.) [69]. The phylogeny of the 64 taxa on which character changes were reconstructed was originally retrieved from Timetree.org [68] and was subsequently adapted to include chikilid caecilians following Kamei et al., 2012 [56]. The node ages in this phylogeny represent the medians of the divergence time estimates inferred from the literature by Timetree.org, except for divergence times within Chikilidae, which we inferred from [56]. Figure 2 shows the reconstruction of several traits associated with toxin resistance under accelerated transformation (Acctran).

### 4.4. Selection Analyses

Selection patterns were examined using the program CodeML in the pamlX (v.1.3.1) software package [70]. Each of the 2 codon data sets (orthosteric site and flanking regions) was split into a caecilian data set (40 species) and a non-caecilian amphibian data set (24 species). The resulting four data sets were used as input files in pamlX to estimate ω values (ratios of non-synonymous over synonymous substitutions) under various selection models. Accompanying trees were derived from the phylogeny used for character reconstruction.

First, we applied one-ratio models to estimate single ω values for caecilians and non-caecilian amphibians. Second, we used branch models to estimate separate ω values for two branch sets in the caecilian tree: (1) a branch set combining all branches that overlapped in time with elapid presence (e.g., including most terminal branches and internal branches overlapping/younger than Early Oligocene for African and Asian taxa, as well as internal branches younger than 30 Mya for American taxa, where elapid snakes arrived later) and (2) branches that did not overlap with elapid presence. These branch sets are illustrated in Supplementary Figure S1. Third, we applied branch models to estimate ω values for five branch sets in the caecilian tree: (1) caecilian branches older than the elapid radiation, (2) Seychelles branches, (3) African branches overlapping with elapid radiation, (4) American branches overlapping with elapid radiation, and (5) Asian branches overlapping with elapid radiation. These branch sets are illustrated in Supplementary Figure S2. All of the above-mentioned analyses were conducted in parallel on the orthosteric-site data set and the flanking-regions data set to evaluate whether inferred patterns of positive selection were restricted to the orthosteric site, as evidence of the true adaptive evolution of toxin resistance. Similarly, to investigate whether patterns of positive selection are restricted to the *chrna1* gene, which encodes the only alpha subunit accessible by alpha-neurotoxins, we conducted selection analyses on homologous codon sets of the genes *chrna2* and *chrna4*. Unlike *chrna1*, we were restricted to species for which *chrna2* and *chrna4* sequences could be retrieved from online databases. Hence, the resulting data sets featured only three caecilian species (*Rhinatrema bivittatum*, *Microcaecilia unicolor*, and *Geotrypetes seraphini*) and five non-caecilian amphibians: (*Ambystoma mexicanum* [Urodela: Ambystomatidae], *Pleurodeles waltl* [Urodela: Salamandridae], *Bufo bufo* [Anura: Bufonidae], *Nanorana parkeri* [Anura: Dicroglossidae], and *Xenopus tropicalis* [Anura: Pipidae]). Finally, to identify specific codons under positive selection in the orthosteric site of American and Asian caecilians, we conducted an analysis with a branch-site model, combining the previously defined Asian and American branch sets (Supplementary Figure S2) in a single foreground branch set (along which sites are allowed to show ω values > 1, marking positive selection) and all other branches (along which sites are only allowed to show ω values ≤ 1, marking purifying selection or neutral evolution).

## Figures and Tables

**Table 1:** Patterns of Darwinian selection in the orthosteric (toxin-binding) site of the alpha-1 subunit and its flanking regions. Listed ω values represent estimated ratios of non-synonymous over synonymous codon substitutions (ω). Values < 1 reflect purifying selection, values close to 1 reflect neutral evolution, and values > 1 (in bold) represent positive selection. The term “N.A.” in the flanking regions of Seychelles caecilians is a result of the lack of sequence divergence within the group.

		ω Values	
Selection Model	Clade/Branch Set	Orthosteric (Toxin-Binding Site)	Flanking Regions
One-ratio model (M0)	non-caecilian amphibians (24 spp.)	0.0852	0.0205
One-ratio model (M0)	caecilians (40 spp.)	0.6019	0.1907
Branch model (M2) with two branch sets	Caecilian branches in the absence of elapids	0.2168	0.0104
	Caecilian branches in the presence of elapids	0.9699	0.452
Branch model (M2) with five branch sets	Caecilian branches older than elapid radiation (Early Oligocene)	0.2411	0.0119
	African caecilians overlapping with elapid radiation	0.1703	0.2322
	American caecilians overlapping with elapid radiation	**1.7789**	0.4039
	Asian Caecilians overlapping with elapid radiation	**2.6121**	0.7442
	Seychelles caecilians (in the absence of elapids)	0.0001	N.A.

**Table 2:** Patterns of Darwinian selection in paralogous receptor subunits.

		ω Values	
Selection Model	Clade/Branch Set	*chrna2*	*chrna4*
One-ratio model (M0)	three caecilian species + five non-caecilian species (eight spp.)	0.0001	0.0015
Branch model (M2)	Caecilians (three spp.)	0.0001	0.0037
	Non-caecilian amphibians (five spp.)	0.0001	0.0012

**Table 3:** Caecilian taxa examined in this study. RGK (Rachunliu G. Kamei) = field voucher number.

Gymnophiona				
Family	Species	Serial Number	Source	Locality
Caeciliidae	*Caecilia abitaguae*	MZUTI 4030	MZUTI	Ecuador
Caeciliidae	*Caecilia guntheri*	MZUTI 3039	MZUTI	Ecuador
Caeciliidae	*Caecilia orientalis*	MZUTI 1371	MZUTI	Ecuador
Caeciliidae	*Caecilia tentaculata*	MZUTI 3919	MZUTI	Ecuador
Chikilidae	*Chikila alcocki*	BNHS 6210 (RGK 0804)	BNHS	India
Chikilidae	*Chikila gaiduwani*	BNHS 6211 (RGK 0102)	BNHS	India
Dermophiidae	*Dermophis mexicanus*	RAN 31534	UMMZ	Guatemala
Dermophiidae	*Geotrypetes seraphini*	XM_033946945.1	GenBank	Cameroon
Dermophiidae	*Gymnopis syntrema*	RAN 31499	UMMZ	Guatemala
Dermophiidae	*Schistometopum thomense*	RAN 31503	UMMZ	São Tomé
Grandisoniidae	*Gegeneophis ramaswamii*	UK MW331	NHM	India
Grandisoniidae	*Hypogeophis alternans*	RAN 31465	UMMZ	Seychelles
Grandisoniidae	*Hypogeophis brevis*	SM 691	NHM	Seychelles
Grandisoniidae	*Hypogeophis cooperi*	RAN 31305	UMMZ	Seychelles
Grandisoniidae	*Hypogeophis larvatus*	SM 297	NHM	Seychelles
Grandisoniidae	*Hypogeophis pti*	SM 295	NHM	Seychelles
Grandisoniidae	*Hypogeophis rostratus*	RAN 31441	UMMZ	Seychelles
Ichthyophiidae	*Ichthyophis garoensis*	BNHS 6208 (RGK 0157)	BNHS	India
Ichthyophiidae	*Ichthyophis orthoplicatus*	DNM MW1723	/	Sri Lanka
Ichthyophiidae	*Ichthyophis sende* *nyu*	BNHS 6209 (RGK 0809)	BNHS	India
Ichthyophiidae	*Uraeotyphlus malabaricus*	UK MW1711	NHM	India
Ichthyophiidae	*Uraeotyphlus oxyurus*	UK MW212	NHM	India
Siphonopidae	*Microcaecilia* sp.	NHM MW995	NHM	Guyana
Siphonopidae	*Siphonops annulatus*	RAN 31968	UMMZ	Ecuador
Siphonopidae	*Siphonops paulensis*	CHUNB 39114	CHUNB	Brazil
Typhlonectidae	*Chthonerpeton indistinctum*	MCP MW15	MCP	Brazil
Typhlonectidae	*Potomotyphlus kaupii*	UMFS 11777	UMMZ	Peru
Typhlonectidae	*Typhlonectes compressicauda*	UMFS 11776	UMMZ	Peru
Typhlonectidae	*Typhlonectes natans*	BMNH 2000.218.	NHM	Colombia

## Data Availability

All data is presented in the paper.

## Supplementary Materials

The supporting information can be downloaded at: https://www.mdpi.com/article/10.3390/ijms241411353/s1.
