# Supplementary material for: Resistance Is Not Futile: Widespread Convergent Evolution of Resistance to Alpha-Neurotoxic Snake Venoms in Caecilians (Amphibia: Gymnophiona)
Source: Int J Mol Sci. 2023 Jul 12;24(14):11353. doi: 10.3390/ijms241411353 (PMC10379402; doi:10.3390/ijms241411353)
Supplement: Supplementary file 1 [file ijms-24-11353-s001.zip › Supplementary Figures S1 and S2.pdf]

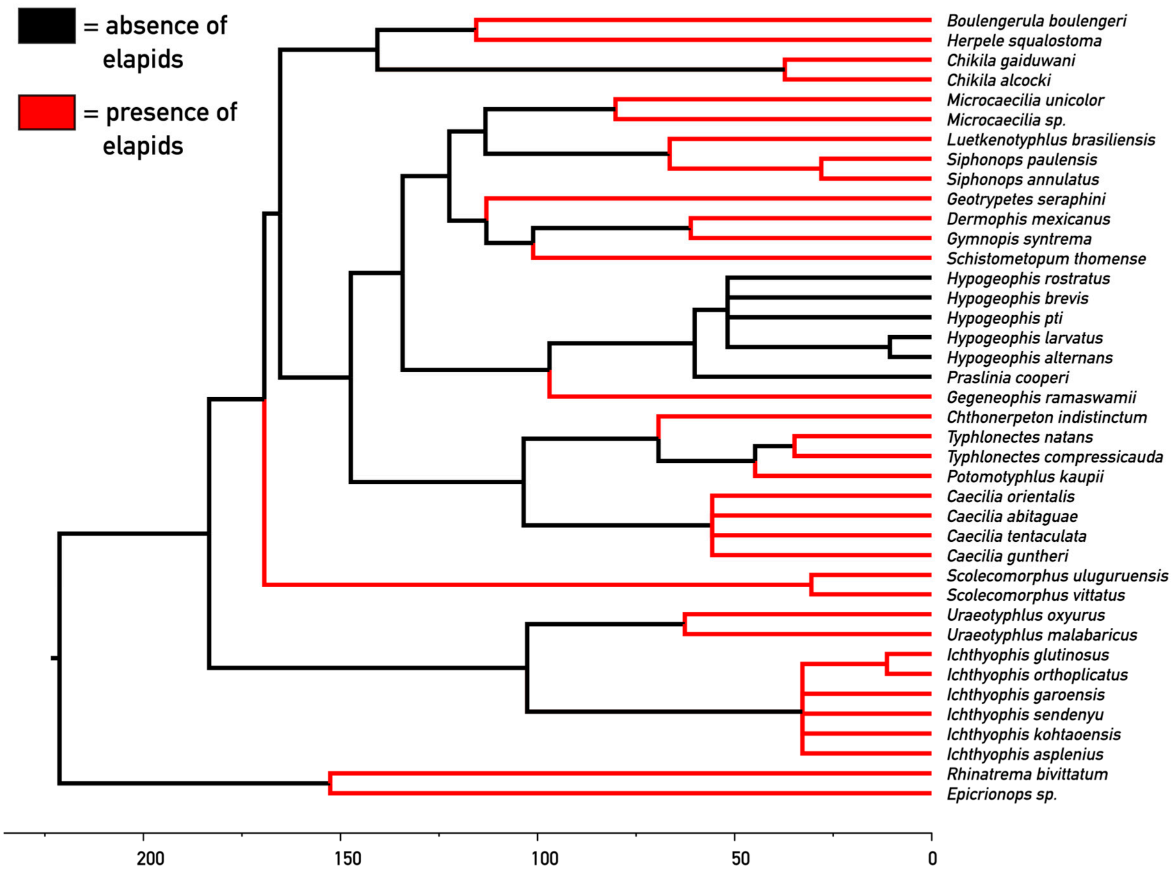

Supplementary Figure S1. showing branches evolving under presence or absence of elapid snakes.

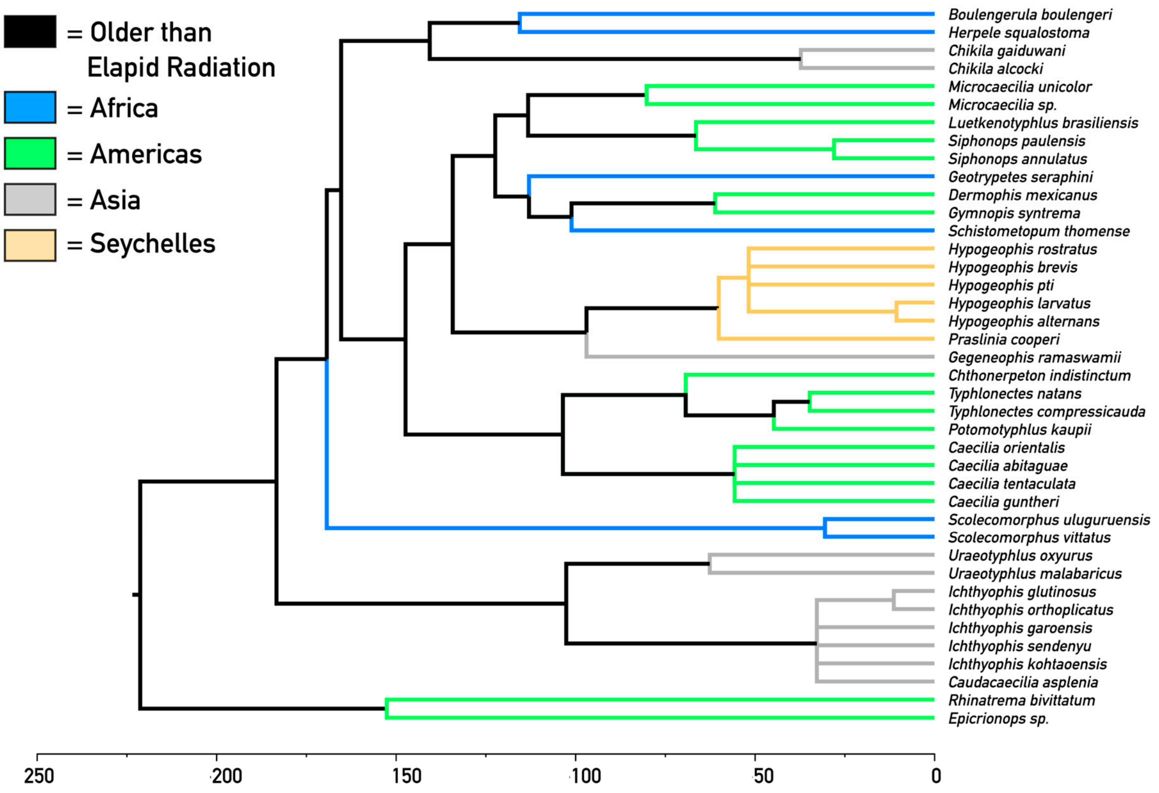

Supplementary Figure S2. showing branches divided by geographical location.
