# Supplementary material for: Resistance Is Not Futile: Widespread Convergent Evolution of Resistance to Alpha-Neurotoxic Snake Venoms in Caecilians (Amphibia: Gymnophiona)
Source: Int J Mol Sci. 2023 Jul 12;24(14):11353. doi: 10.3390/ijms241411353 (PMC10379402; doi:10.3390/ijms241411353)
Supplement: Supplementary file 1 [file ijms-24-11353-s001.zip › Supplementary File S1.pdf]

Chrna1 - orthosteric region (alpha-1 positions 187-200)
>NC_044037.1:223677047-223677226 Microcaecilia unicolor chromosome 7, aMicrocaecilia unicolor1.1, whole genome shotgun sequence
CGCGTAGACTACTCCTGCTGTCCGGACACTCCGTATCTGGAT
>NC_042620.1:115850301-115850480 Rhinatrema bivittatum chromosome 6, aRhinatrema bivittatum1.1, whole genome shotgun sequence
CGGGTGAACTACTCCTGCTGTGGAGATACTCCATACCTGGAT
>Caudacaecilia asplenia_ZRC1.10835
GACATATACTATTCCTGCTGTCCGGATACTCCCTATCTGGAT
>Ichthyophis glutinosus_WHT5508
TACGTAAACTATAGCTGCTGTCCGGACACTCCCTACCTGGAT
>Ichthyophis kohtaoensis
AACATATCCTATGCCTGCTGTCTGGAGACTCCCTATCTGGAT
>Ichthyophis garoensis_BNHS2208
AACATATCCTATGCCTGCTGTCCGGAAACTCCCTATCTGGAT
>Ichthyophis sendenyu_BNHS2209
AACATATCCTATGCCTGCTGTCTGGAAACTCCCTATCTGGAT
>Ichthyophis orthoplicatus_DNMMW1723
TACGTAAACTATAGCTGCTGTCCGGACACTCCCTACCTGGAT
>Uraeotyphlus cf. malabaricus_UKMW1711
TACGTAACCTATGCCTGCTGTCAGGACACTCCCTATCTGGAT
>Uraeotyphlus cf. oxyurus_UK MW212
TACGTAATCTATGCCTGCTGTCTGGAAACTCCCTATCTGGAT
>Scolecomorphus uluguruensis_BMNH2002.099
TATGTATACTACGCCTGCTGTCAGAACACTCCATATCTGGAT
>Scolecomorphus vittatus CAS168810
TACGTATACTACGCCTGCTGTCAGAACACTCCGTATCTGGAT
>Herpele squalostoma_BMNH2000.096
TACGTGTACTATGCCTGCTGTCCGAACACGCCGTACCTGGAC
>Boulengerula boulengeri_RAN31530
TACGTGTACTACGCCTGCTGTCTAGAAACTCCATACCTGGAC
>Chthonerpeton indistinctum_MCPMW15
TACGTGCACTACGCCTGCTGTCCAGACACTCCTTATCTGGAC
>Typhlonectes compressicauda_UMFS11776
TATGTGACTTACTCCTGCTGTCCAGATACTCCTTATCTGGAT
>Typhlonectes natans_BMNH2000.218.
TATGTGAACTACTCCTGCTGTCCAGATACTCCTTATCTGGAT
>Potomotyphlus kaupii_UMFS11777
TATGTGACTTACTCCTGCTGTCCAGATACTCCTTATCTGGAT
>Caecilia abitaguae_MZUTI4030
TACGTGACCTACGCCTGCTGTCTAGACACTCCTTATCTGGAT
>Caecilia guntheri_MZUTI3039
AACGTGACCTACGGCTGCTGTCTAGACACTCCTTATCTGGAT
>Caecilia orientalis_MZUTI1371
TACGTGACCTACGCCTGCTGTCCAGACACTCCTTATCTGGAT
>Caecilia tentaculata_MZUTI3919
AACGTGACCTACTCCTGCTGTGGAGACACTCCTTATCTGGAT

>Gegeneophis ramaswamii_UKMW331
TACATAAACTACAGCTGCTGTCCAGACGTTCCGTATCTGGAT
>Hypogeophis rostratus_RAN31441
TACGTAACCTACGCCTGCTGTCCAGACCTTCCATATCTGGAT
>Praslinia cooperi_RAN31305
TACGTAACCTACGCCTGTTGTCCAGACCTTCCATATCTGGAT
>Hypogeophis alternans_RAN 31465
TACGTAACCTACGCCTGCTGTCCAGACCTTCCATATCTGGAT
>Luetkenotyphlus brasiliensis_BMNH2005.3
TATGTAACCTACGCCTGCTGTCCGGACACTCCGTATCTGGAT
>Microcaecilia sp,_NHMMW995
TATGTAACCTACGCTTGCTGTCCGAACACTCCGTATCTGGAT
>Siphonops annulatus_RAN31968
TGTGTAACCTACTCCTGCTGTCTGGACACTCCGTATCTGGAT
>Siphonops paulensis_CHUNB39114
TGTGTAACCTACGCCTGCTGTCAGGACACTCCGTATCTGGAT
>Schistometopum thomense_RAN31503
AACGTAACCTACGGCTGCTGTCCAGACACTCCCTATTTGGAT
>Geotrypetes_seraphini_XM_033939894.1
TACGTCACCTATGCCTGTTGTCCGGACACTCCATATCTGGAT
>Dermophis mexicanus_RAN31534
TTCGTATGCTACTCCTGCTGTCCAGACACTCCATATCTGGAT
>Gymnopis syntrema_RAN31499
TACRTASMSTACGGCTGCTGTGCAGACACTCTGTATCTGGAT
>Chikila gaiduwani_BNHS2211
AACGTGACGTACTTCTGCTGTGGGGACACTCCGTATCTGGAC
>Chikila alcocki_BNHS2210
AACGTGACGTACGCCTGCTGTGGGGACACTCCGTATCTGGAC
>Hypogeophis pti_SM295
NNNNNNNCCTACGCCTGCTGTCCAGACCTTCCATATCTGGAT
>Hypogeophis larvatus_SM297
NNNNNNACATACGCCTGCTGTCCAGACCTTCCATATCTGGAT
>Hypogeophis brevis_SM691
NNNNNNNCCTACGCCTGCTGTCCAGACCTTCCATATCTGGAT
>CM030321.1:437425379-437425558 Ambystoma mexicanum strain DD151 chromosome 9q, whole genome shotgun sequence
TGGGTTTATTATACCTGCTGTCCGGATACACCATACTTGGAC
>M0365642_Pleurodeles_waltl_PLEWA04_M0365642_PLEWA02_M0365642_PLEWA02 TRINITY_DN300233_c9_g1::TRINITY_DN300233_c9_g1_i1::g.365642::m.365642 TRINITY_DN300233_c9_g1::TRINITY_DN300233_c9_g1_i1::g.365642 ORF type:5prime_partial len:487 (+) TRINITY_DN300233_c9_g1_i1:1-1461(+) parent_contig=Trinity_Pw_v2
TGGGTGTATTATGCATGTTGTCAGGAAACACCATACTTGGAC
>CAJPDP013104969.1:36-215 Bombina variegata variegata genome assembly, contig: contig_3104969, whole genome shotgun sequence
TGGGTTTATTACGCCTGCTGTCCTGACACCCCATATCTGGAT
>LR991673.1:183921276-183921455 Bufo bufo genome assembly, chromosome: 7
TGGGTCACATACACATGCTGTCCTGATGTACCATACTTGGAT

>CM026472.1:34540173-34540352 Bufo gargarizans isolate SCDJY-AF-19 chromosome 8, whole genome shotgun sequence
TGGGTCACATACACATGCTGTCTTAACGTACCATACTTGGAT
>CM033648.1:76491222-76491401 Engystomops pustulosus isolate 237g6f4 chromosome 8, whole genome shotgun sequence
TATGTTTTCTATGACTGCTGCCCTGATAAACCATATCTGGAT
>ONZH01026881.1:670711-670890 Rhinella marina genome assembly, contig: ctg3915_RHIMB__RM170330.3915, whole genome shotgun sequence
GGGGTCGAATACACATGCTGTCCTGACGTCCCATACTTGGAT
>VIAB01000030.1:258890-259069 Oophaga pumilio Sca30, whole genome shotgun sequence
TGGGTCACATATGCCTGCTGCCCCGATGTCCCATACTTGGAT
>CM034095.1:51234791-51234970 Eleutherodactylus coqui isolate HN-11 Male chromosome 10, whole genome shotgun sequence
TGGGTCACATATGCGTGCTGTCCGGATGTCCCATACTTGGAT
>KN905892.1:795302-795481 Nanorana parkeri isolate BGI_ZX_2015 unplaced genomic scaffold scaffold44, whole genome shotgun sequence
TGGGTTTTCTATACCTGCTGTCCTGACGTTCCATACCTGGAC
>JAEOBE010700063.1:713-892 Phrynoglossus myanhessei voucher SMF 103841 isolate Holotype NODE_4282317_length_3454_cov_14.630863, whole genome shotgun sequence
TGGGTTTACTATGGTTGCTGTCCTGATGTCCCATACCTGGAC
>WWET01001398.1:49535-49714 Limnodynastes dumerilii isolate SAMAR66870 scaffold001398, whole genome shotgun sequence
TGGGTCACATACACATGCTGCCCTGATGTCCCCTACTTAGAT
>JABWIB010022546.1:4978-5157 Platyplectrum ornatum isolate SVE-2020 scaffold_22545, whole genome shotgun sequence
TGGGTTACATACACGTGCTGTCCTGATGTTCCATACCTGGAT
>CM032236.1:50885104-50885283 Leptobrachium ailaonicum isolate 2019-ailao-v1_NWPU chromosome 3, whole genome shotgun sequence
TGGGTTTATTATGACTGCTGCCTTGATACGCCTTACCTGGAT
>CM033477.1:122732597-122732776 Hymenochirus boettgeri isolate Female2 chromosome 9, whole genome shotgun sequence
TGGGTGTATTATGACTGCTGCCCGGAAACACCATATCTAGAC
>JAKJJM010035931.1:697-876 Pipa carvalhoi isolate CFBHT 2225 Pipa_carvalhoi_contig_35931, whole genome shotgun sequence
TGGGTTTATTATGATTGTTGTCCTGATACACCATACCTGGAT
>CM030356.1:77401314-77401493 Xenopus laevis strain J_2021 chromosome 9_10L, whole genome shotgun sequence
TGGGTTTATTATGACTGCTGCCCAGAAACACCATACTTGGAT
>CM004451.2:66280523-66280702 Xenopus tropicalis strain Nigerian chromosome 9, whole genome shotgun sequence
TGGGTTTATTATGACTGTTGCCCAGAAACGCCATACTTGGAT
>CM016422.1:54052159-54052338 Pyxicephalus adspersus isolate 1538 chromosome 7, whole genome shotgun sequence
TGGGTTTGTTATTCTTGTTGTCCAGACGTTCCATACCTGGAC
>BLSH010491879.1:618965-619144 Glandirana rugosa DNA, scaffold10449653_len1176436_cov66_read141_ma xK112, whole genome shotgun sequence

TGGGTGGTCTATGACTGCTGCCCTGACACACCATACTTGGAT
>LR991685.1:163324039-163324218 Rana temporaria genome assembly, chromosome: 6
TGGGTCGTCTATGACTGCTGTCCTGACACACCATACTTGGAT
>KV992222.1:13352-13531 Lithobates catesbeianus isolate Bruno unplaced genomic scaffold Rc-01r160223s0069667, whole genome shotgun sequence
TGGGTCGTCTATGACTGCTGTCCTGACACACCATACTTGGAT
>VKOB010690764.1:40-219 Scaphiopus holbrookii voucher NCSM:84231 6967497, whole genome shotgun sequence
TGGGTTTATTATACCTGCTGCCCCGATAAGCCATACCTAGAC
>VKNZ010744601.1:18-197 Spea bombifrons voucher NCSM:84228 4498154, whole genome shotgun sequence
TGGGTTTATTATGACTGCTGTCCAACCAAACCGTACCTGGAC

Chrna1 - alpha-1 positions 180-186
>CM030321.1:437425379-437425558 Ambystoma mexicanum strain DD151 chromosome 9q, whole genome shotgun sequence
GATTACCGAGGTTGGAAACAC
>CAJPDP013104969.1:36-215 Bombina variegata variegata genome assembly, contig: contig_3104969, whole genome shotgun sequence
GACAACCGCTGTTGGAAGCAT
>LR991673.1:183921276-183921455 Bufo bufo genome assembly, chromosome: 7
GATTACCGCTGCTGGAAACAC
>CM026472.1:34540173-34540352 Bufo gargarizans isolate SCDJY-AF-19 chromosome 8, whole genome shotgun sequence
GATTACCGCTGCTGGAAACAC
>CM034095.1:51234791-51234970 Eleutherodactylus coqui isolate HN-11 Male chromosome 10, whole genome shotgun sequence
GATTACCGCTGCTGGAAACAT
>CM033648.1:76491222-76491401 Engystomops pustulosus isolate 237g6f4 chromosome 8, whole genome shotgun sequence
GATTACCAATGCTGGAAGCAC
>BLSH010491879.1:618965-619144 Glandirana rugosa DNA, scaffold10449653_len1176436_cov66_read141_ma xK112, whole genome shotgun sequence
GATTATCAATGCTGGAAGCAT
>CM033477.1:122732597-122732776 Hymenochirus boettgeri isolate Female2 chromosome 9, whole genome shotgun sequence
GATTATCGTTGCTGGAAGCAC
>CM032236.1:50885104-50885283 Leptobrachium ailaonicum isolate 2019-ailao-v1_NWPU chromosome 3, whole genome shotgun sequence
GATTACCGCTGCTGGAAACAC
>WWET01001398.1:49535-49714 Limnodynastes dumerilii isolate SAMAR66870 scaffold001398, whole genome shotgun sequence
GATTATCAGTGCTGGAAGCAC
>KV992222.1:13352-13531 Lithobates catesbeianus isolate Bruno unplaced genomic scaffold Rc-01r160223s0069667, whole genome shotgun sequence
GATTATCAATGCTGGAAGCAT
>KN905892.1:795302-795481 Nanorana parkeri isolate BGI_ZX_2015 unplaced genomic

scaffold scaffold44, whole genome shotgun sequence
GATTATCAGTGCTGGAAGCAT
>VIAB01000030.1:258890-259069 Oophaga pumilio Sca30, whole genome shotgun sequence
GATTACCGCTGCTGGAAGCAT
>JAEOBE010700063.1:713-892 Phrynoglossus myanhessei voucher SMF 103841 isolate Holotype NODE_4282317_length_3454_cov_14.630863, whole genome shotgun sequence
GATTATCAATGTTGGAAGCAT
>JAKJJM010035931.1:697-876 Pipa carvalhoi isolate CFBHT 2225 Pipa_carvalhoi_contig_35931, whole genome shotgun sequence
GACTATCGATGCTGGAAACAC
>JABWIB010022546.1:4978-5157 Platyplectrum ornatum isolate SVE-2020 scaffold_22545, whole genome shotgun sequence
GACTATCGCTGCTGGAAGCAT
>M0365642_Pleurodeles_waltl_PLEWA04_M0365642_PLEWA02_M0365642_PLEWA02 TRINITY_DN300233_c9_g1::TRINITY_DN300233_c9_g1_i1::g.365642::m.365642 TRINITY_DN300233_c9_g1::TRINITY_DN300233_c9_g1_i1::g.365642 ORF type:5prime_partial len:487 (+) TRINITY_DN300233_c9_g1_i1:1-1461(+) parent_contig=Trinity_Pw_v2
GATTACCGAGGTTGGAAACAC
>CM016422.1:54052159-54052338 Pyxicephalus adspersus isolate 1538 chromosome 7, whole genome shotgun sequence
GATTATCGCTGTTGGAAACAC
>LR991685.1:163324039-163324218 Rana temporaria genome assembly, chromosome: 6
GATTATCAATGCTGGAAGCAT
>ONZH01026881.1:670711-670890 Rhinella marina genome assembly, contig: ctg3915_RHIMB__RM170330.3915, whole genome shotgun sequence
GATTACCGCTGCTGGAAACAT
>VKOB010690764.1:40-219 Scaphiopus holbrookii voucher NCSM:84231 6967497, whole genome shotgun sequence
GACTATCGATGCTGGAAGCAT
>VKNZ010744601.1:18-197 Spea bombifrons voucher NCSM:84228 4498154, whole genome shotgun sequence
GATTATCGATGCTGGAAGCAT
>CM030356.1:77401314-77401493 Xenopus laevis strain J_2021 chromosome 9_10L, whole genome shotgun sequence
GATTATCGCTGCTGGAAGCAC
>CM004451.2:66280523-66280702 Xenopus tropicalis strain Nigerian chromosome 9, whole genome shotgun sequence
GATTATCGTGGCTGGAAGCAC
>Epicrionops sp._UMMZRAN26114
GATTACCAGGGCTGGAAGCAC
>Rhinatrema bivittatum_XM_029605825.1
GATTACAAGAGCTGGAAGCAC
>Caudacaecilia asplenia_ZRC1.10835
GATTACCGAGGCTGGAAACAC
>Ichthyophis kohtaoensis_
GATTGCCAAGGCTGGAAACAC

>Ichthyophis garoensis_BNHS2208
GATTACCGAGGCTGGAAACAC
>Ichthyophis sendenyu_BNHS2209
GATTACCGAGGCTGGAAACAC
>Ichthyophis orthoplicatus_DNMMW1723
GATTACCGAGGCTGGAAGCAC
>Ichthyophis glutinosus_WHT5508
GATTACCGAGGCTGGAAGCAC
>Uraeotyphlus cf. malabaricus_UKMW1711
GATTACCGAGGCTGGAAACAC
>Uraeotyphlus cf. oxyurus_UK MW212
GATTGCAGAGGCTGGAAACAC
>Scolecomorphus uluguruensis_BMNH2002.099
GATTACCGAGGCTGGAAGCAT
>Scolecomorphus vittatus_CAS168810
GATTACAGAGGCTGGAAGCAT
>Herpele squalostoma_BMNH2000.096
GATTACCGAGGCTGGAAGCAT
>Boulengerula boulengeri_RAN31530
GATTACCGAAGCTGGAAGCAT
>Chthonerpeton indistinctum_MCPMW15
GATTACCGATGCTGGAAGCAT
>Typhlonectes compressicauda_UMFS11776
GATTACCGAGGCTGGAAGCAT
>Typhlonectes natans_BMNH2000.218.
GATTACCGAGGCTGGAAGCAT
>Potomotyphlus kaupii_UMFS11777
GATTACMGAGGCTGGAAGCAT
>Caecilia abitaguae_MZUTI4030
GATTACCGAGGCTGGAAGCAT
>Caecilia guntheri_MZUTI3039
GATTACCGAGGCTGGAAGCAT
>Caecilia orientalis_MZUTI1371
GATTACCGAGGCTGGAAGCAT
>Caecilia tentaculata_MZUTI3919
GATTACCGAGGCTGGAAGCAT
>Gegeneophis ramaswamii_UKMW331
GATTACCGAGGCTGGAAGCAT
>Hypogeophis rostratus_RAN31441
GATTACCGAGGCTGGAAGCAT
>Praslinia cooperi_RAN31305
GATTACCGAGGCTGGAAGCAT
>Hypogeophis alternans_RAN 31465
GATTACCGAGGCTGGAAGCAT
>Luetkenotyphlus brasiliensis_BMNH2005.3
GATTACCGAGGCTGGAAGCAT
>Microcaecilia sp,_NHMMW995

GATTACCGAGGCTGGAAGCAT
>Microcaecilia unicolor_XM_030209957.1
GATTACCGAGGCTGGAAGCAT
>Siphonops annulatus_RAN31968
GATTACCGAGGCTGGAAGCAT
>Siphonops paulensis_CHUNB39114
GATTACCGAGGCTGGAAGCAT
>Schistometopum thomense_RAN31503
GATTACCGAGGCTGGAGGCAT
>Geotrypetes seraphini_XM_033946945.1
GATTACCGAGGCTGGAAGCAT
>Dermophis mexicanus_RAN31534
GATTACCGAGGGTGGAAGCAT
>Gymnopis syntrema_RAN31499
GATTACCGAGGCTGGAAGCAT
>Chikila alcocki_BNHS2210
GATTTCCAGGGCTGGCCGCAT
>Chikila gaiduwani_BNHS2211
GATTTCCAGGGCTGGCCGCAT
>Hypogeophis pti_SM295

>Hypogeophis brevis_SM691

>Hypogeophis larvatus_SM297

Chrna1 - alpha-1 positions 201-207
>CM030321.1:437425379-437425558 Ambystoma mexicanum strain DD151 chromosome 9q, whole genome shotgun sequence
ATTACATATCATTTCCTCATG
>CAJPDP013104969.1:36-215 Bombina variegata variegata genome assembly, contig: contig_3104969, whole genome shotgun sequence
ATCACATATCACTTCCTTATG
>LR991673.1:183921276-183921455 Bufo bufo genome assembly, chromosome: 7
GTCACCTACCACTTCGTCCTA
>CM026472.1:34540173-34540352 Bufo gargarizans isolate SCDJY-AF-19 chromosome 8, whole genome shotgun sequence
GTCACCTACCACTTCGTCCTA
>CM034095.1:51234791-51234970 Eleutherodactylus coqui isolate HN-11 Male chromosome 10, whole genome shotgun sequence
ATCACCTACCACTTCGTCTTA
>CM033648.1:76491222-76491401 Engystomops pustulosus isolate 237g6f4 chromosome 8, whole genome shotgun sequence
ATCACCTACCATTTCCTTCTG
>BLSH010491879.1:618965-619144 Glandirana rugosa DNA, scaffold10449653_len1176436_cov66_read141_ma xK112, whole genome shotgun sequence
ATCACCTATCATTTCCTTATG

>CM033477.1:122732597-122732776 Hymenochirus boettgeri isolate Female2 chromosome 9, whole genome shotgun sequence
ATCACATACCATTTCCTCTTG
>CM032236.1:50885104-50885283 Leptobrachium ailaonicum isolate 2019-ailao-v1_NWPU chromosome 3, whole genome shotgun sequence
ATCACCTACCACTTCCTCTTG
>WWET01001398.1:49535-49714 Limnodynastes dumerilii isolate SAMAR66870 scaffold001398, whole genome shotgun sequence
ATCACCTTCCATTTCCTCCTG
>KV992222.1:13352-13531 Lithobates catesbeianus isolate Bruno unplaced genomic scaffold Rc-01r160223s0069667, whole genome shotgun sequence
ATCACCTATCATTTCCTCATG
>KN905892.1:795302-795481 Nanorana parkeri isolate BGI_ZX_2015 unplaced genomic scaffold scaffold44, whole genome shotgun sequence
ATCACCTATCATTTCCTCATG
>VIAB01000030.1:258890-259069 Oophaga pumilio Sca30, whole genome shotgun sequence
ATCACCTACCATTTCGTCTTA
>JAEOBE010700063.1:713-892 Phrynoglossus myanhessei voucher SMF 103841 isolate Holotype NODE_4282317_length_3454_cov_14.630863, whole genome shotgun sequence
ATCACCTATCATTTCCTGATG
>JAKJJM010035931.1:697-876 Pipa carvalhoi isolate CFBHT 2225 Pipa_carvalhoi_contig_35931, whole genome shotgun sequence
ATCACTTACCACTTTCTCTTA
>JABWIB010022546.1:4978-5157 Platyplectrum ornatum isolate SVE-2020 scaffold_22545, whole genome shotgun sequence
ATCACCTATCACTTCGTCTTG
>M0365642_Pleurodeles_waltl_PLEWA04_M0365642_PLEWA02_M0365642_PLEWA02 TRINITY_DN300233_c9_g1::TRINITY_DN300233_c9_g1_i1::g.365642::m.365642 TRINITY_DN300233_c9_g1::TRINITY_DN300233_c9_g1_i1::g.365642 ORF type:5prime_partial len:487 (+) TRINITY_DN300233_c9_g1_i1:1-1461(+) parent_contig=Trinity_Pw_v2
ATCACATATCATTTCCTCATG
>CM016422.1:54052159-54052338 Pyxicephalus adspersus isolate 1538 chromosome 7, whole genome shotgun sequence
ATAACCTATCACTTCATACTG
>LR991685.1:163324039-163324218 Rana temporaria genome assembly, chromosome: 6
ATCACCTATCATTTCCTCATG
>ONZH01026881.1:670711-670890 Rhinella marina genome assembly, contig: ctg3915_RHIMB__RM170330.3915, whole genome shotgun sequence
ATCACCTACCACTTCGTCCTA
>VKOB010690764.1:40-219 Scaphiopus holbrookii voucher NCSM:84231 6967497, whole genome shotgun sequence
ATCACCTACCACTTTATCCTA
>VKNZ010744601.1:18-197 Spea bombifrons voucher NCSM:84228 4498154, whole genome shotgun sequence
ATCACCTACCACTTTGTGCTG
>CM030356.1:77401314-77401493 Xenopus laevis strain J_2021 chromosome 9_10L, whole

genome shotgun sequence
ATCACATACCATTTTCTCCTG
>CM004451.2:66280523-66280702 Xenopus tropicalis strain Nigerian chromosome 9, whole genome shotgun sequence
ATCACATACCATTTTCTCCTG
>Epicrionops sp._UMMZRAN26114
ATCACTTACCATTTCCTGATG
>Rhinatrema bivittatum_XM_029605825.1
ATCACTTACCACTTCCTGTTG
>Caudacaecilia asplenia_ZRC1.10835
ATCACCTACCATTTCGTAATG
>Ichthyophis kohtaoensis_
ATCACCTACCATTTCCTAATG
>Ichthyophis garoensis_BNHS2208
ATCACCTACCATTTCC-----
>Ichthyophis sendenyu_BNHS2209
ATCACCTACCATTTC------
>Ichthyophis orthoplicatus_DNMMW1723
ATCACCTACCATTTCCTGATG
>Ichthyophis glutinosus_WHT5508
ATCACCTACCATTTCCTGATG
>Uraeotyphlus cf. malabaricus_UKMW1711
ATCACTTACCATTTC-TGATG
>Uraeotyphlus cf. oxyurus_UK MW212
ATCACCTACCATTTCGTGATG
>Uraeotyphlus cf. malabaricus_UKMW1711
ATCACTTACCATTTC-TGATG
>Scolecomorphus uluguruensis_BMNH2002.099
ATCACCTACCACTTCCTGATG
>Scolecomorphus vittatus_CAS168810
ATCACCTACCACTCCCTGATG
>Herpele squalostoma_BMNH2000.096
ATCACATACCACTTCCTGATG
>Boulengerula boulengeri_RAN31530
ATCACGTACCACTTCCTGATG
>Chthonerpeton indistinctum_MCPMW15
ATCACTTACCACTTCCTGATG
>Typhlonectes compressicauda_UMFS11776
ATCACTTACCACTTTCTGATG
>Typhlonectes natans_BMNH2000.218.
ATCACTTACCACTTCCTGATG
>Potomotyphlus kaupii_UMFS11777
ATCACTTACCACTTCTTGATG
>Caecilia abitaguae_MZUTI4030
ATCACTTATCACTTCCTGATG
>Caecilia guntheri_MZUTI3039
ATCACTTATCACTTCCTGTTG

>Caecilia orientalis_MZUTI1371
ATCACTTATCACTTCCTGATG
>Caecilia tentaculata_MZUTI3919
ATCACTTATCACTTGCTGTTG
>Gegeneophis ramaswamii_UKMW331
ATCACTTATCACTTCGTGCTG
>Hypogeophis rostratus_RAN31441
ATCACTTATCACTTCCTGATG
>Praslinia cooperi_RAN31305
ATCACTTATCACTTCCTGATG
>Hypogeophis alternans_RAN 31465
ATCACTTATCACTTCCTGATG
>Luetkenotyphlus brasiliensis_BMNH2005.3
ATCACTTATCACTTCCTGATG
>Microcaecilia sp,_NHMMW995
ATCACTTATCACTTCCTGATG
>Microcaecilia unicolor_XM_030209957.1
ATCACTTATCACTTCCTGATG
>Siphonops annulatus_RAN31968
ATCACTTATCACTTCCTGATG
>Siphonops paulensis_CHUNB39114
ATCACTTATCACTTCCTGATG
>Schistometopum thomense_RAN31503
ATCACTTATCACTTCCTGATG
>Geotrypetes seraphini_XM_033946945.1
ATCACGTATCACTTCCTGATG
>Dermophis mexicanus_RAN31534
ATCACTTATCACTTCCTGATG
>Gymnopis syntrema_RAN31499
ATCACTTATCACTTCCTGATG
>Chikila_alcocki_BNHS2210
ATCACCTACCACTT-------
>Chikila gaiduwani_BNHS2211
ATCACCTACCACTT-------
>Hypogeophis pti_SM295
ATCACTTATCACTTCC-----
>Hypogeophis brevis_SM691
ATCACTTATCACTTCC-----
>Hypogeophis larvatus_SM297
ATCACTTATCACTTCC-----

Chrna2
>Pleurodeles_waltl_/
ACCAAGAAGTATGACTGCTGCACGGAGATCTACCCGGACATC
>Ambystoma_mexicanum_SuperContig_183730
ACCAAGAAGTACGACTGCTGCACTGAGATCTACCCTGATATC
>Bufo_bufo_XM_040427115.1

ACTAAAAAGTATGATTGCTGCACTGAGATTTACCCTGACATC
>Nanorana_parkeri_XM_018570301.1
TCTAAGAAATATGATTGCTGCACGGAAATTTACCCTGATATT
>Xenopus_tropicalis_XM_004914941.4
TCCAAGAAATACGACTGTTGCACGGAAATCTATCCAGATATC
>Rhinatrema_bivittatum_XM_029596210.1
ACTAAAAAGTATGACTGCTGCACTGAAATCTACCCAGACATC
>Microcaecilia_unicolor_XM_030195550.1
ACTAAAAAGTATGATTGCTGCACTGAAATCTACCCAGACATC
>Geotrypetes_seraphini_XM_033939894.1
ACCAAAAAGTATGATTGCTGCACTGAAATCTACCCTGACATCù

Chrna4
>Pleurodeles_waltl_/
ATCAAGAAGTATGAGTGCTGTACTGAGATATACCCTGACATC
>Ambystoma_mexicanum_SuperContig_16675
ACCAAAAAGTATGATTGCTGCACTGAAATCTACCCTGACATC
>Bufo_bufo_XM_040435648.1
ATCAAAAAGTATGAGTGCTGCACAGAAATATACTCAGACATC
>Nanorana_parkeri_XM_018560101.1
ATCAAAAAGTACGAGTGCTGCACGGAGATCTACTCGGACATC
>Xenopus_tropicalis_NM_001113843.1
ATCAAGAAATATGAGTGTTGCACGGAGATTTATTCCGACATC
>Rhinatrema_bivittatum_XM_029613401.1
ACAAAGAAATACGAGTGCTGCACGGAAATCTACCCCGACATC
>Microcaecilia_unicolor_XM_030212004.1
ATTAAGAAATACGAGTGTTGTACTGAAATTTACCCTGATATT
>Geotrypetes_seraphini_XM_033939894.1
ATTAAGAAATATGAGTGCTGCACTGAAATCTACCCTGATATT
